# QAC Resistance Genes in ESBL-Producing *E. coli* Isolated from Patients with Lower Respiratory Tract Infections in the Central Slovenia Region—A 21-Year Survey

**DOI:** 10.3390/tropicalmed8050273

**Published:** 2023-05-12

**Authors:** Katja Hrovat, Jerneja Čremožnik Zupančič, Katja Seme, Jerneja Ambrožič Avguštin

**Affiliations:** 1 Department of Biology, Biotechnical Faculty, University of Ljubljana, 1000 Ljubljana, Slovenia; katja.hrovat@bf.uni-lj.si (K.H.); jerneja.zupancic@bf.uni-lj.si (J.Č.Z.); 2 Institute of Microbiology and Immunology, Faculty of Medicine, University of Ljubljana, 1000 Ljubljana, Slovenia; katja.seme@mf.uni-lj.si

**Keywords:** *Escherichia coli*, lower respiratory tract samples, extended-spectrum β-lactamases, QAC resistance genes

## Abstract

Biocidal products prevent the spread of pathogenic microorganisms, including extended-spectrum β-lactamase-producing *Escherichia coli* (ESBL-EC), which is one of the most alarming health problems worldwide. Quaternary ammonium compounds (QACs) are surface-active agents that interact with the cytoplasmic membrane and are widely used in hospitals and food processing environments. A collection of 577 ESBL-EC, isolated from lower respiratory tract (LRT) samples, was screened for QAC resistance genes *oqxA*; *oqxB*; *qacEΔ1*; *qacE*; *qacF/H/I*; *qacG*; *sugE* (p); *emrE; mdfA; sugE* (c)*; ydgE; ydgF*; and for class 1, 2, and 3 integrons. The prevalence of chromosome-encoded genes ranged from 77 to 100%, while the prevalence of QAC resistance genes encoded on mobile genetic elements (MGEs) was relatively low (0–0.9%), with the exception of *qacEΔ1* (54.6%). PCR screening detected the presence of class 1 integrons in 36.3% (n = 210) of isolates, which were positively correlated with *qacEΔ1*. More correlations between QAC resistance genes, integrons, sequence type group ST131, and β-lactamase genes were presented. The results of our study confirm the presence of QAC resistance genes and also class 1 integrons commonly found in multidrug-resistant clinical isolates and highlight the potential role of QAC resistance genes in the selection of ESBL-producing *E. coli* in hospitals.

## 1. Introduction

*Escherichia coli* (*E. coli*) is a commensal in the intestines of humans and warm-blooded animals but can also cause a variety of intestinal and extraintestinal infections (ExPEC). ExPEC strains are among the most common bacterial pathogens isolated from clinical specimens and are associated with different types of infections, including bacteremia, urinary tract infections, neonatal meningitis, respiratory tract infections, and skin and soft tissue infections [1,2,3,4]. Despite the plasticity and variability of the *E. coli* genome, several studies have shown that ExPEC strains possess a distinct set of virulence-associated genes (VAGs), including adhesins, autotransporters, toxins, siderophores, and protectins, which are usually encoded on MGEs [5,6,7]. These strains mainly belong to phylogenetic group B2 or D with the most frequent sequence type 131 (ST131), which can be found in the community, hospitals, and also in the environment [8,9]. The clonal group ST131 plays an important role in the worldwide distribution of antibiotic-concerning resistance genes in *E. coli*, e.g., resistance to β-lactams and fluoroquinolones. Genes for extended-spectrum β-lactamases (ESBLs) and fluoroquinolone-resistance are often encoded on the same MGEs, with a common combination of *bla*_CTX-M-15_ and *aac(6′)-Ib-cr* [9,10,11,12,13]. The emergence and increasing dissemination of highly virulent and multidrug-resistant ESBL-producing *E. coli* (ESBL-EC) contributes to treatment failure and increased mortality. Therefore, the World Health Organization has classified ESBL-EC in the group of critical pathogens for which research and development of new antibiotics are urgently needed [14]. In order to reduce infections with antimicrobial-resistant microorganisms, effective disinfection and hygiene strategies have been applied in the last two decades, leading to increased use of biocides, which peaked during the SARS-CoV-2 pandemic [15,16].

Biocidal products are of great importance for the control and elimination of pathogens, especially in settings such as hospitals, the food industry, and, recently, increasingly in the domestic environment. In hospitals, biocides are used for medical devices and surface disinfection and also for skin antisepsis. Effective disinfection of hospital surfaces, instruments, and rooms is especially crucial for intensive care patients, where nosocomial infections are often associated with mechanical ventilation [17,18]. Quaternary ammonium compounds (QACs) are cationic surfactants that interact with the cytoplasmic membrane of bacteria, resulting in cell lysis, and are commonly used as biocidal agents. They act on a wide range of microorganisms, including fungi, bacteria, parasites, and lipophilic viruses [16,19]. Continuous exposure to biocidal products exerts constant selective pressure on bacteria and, over time, promotes tolerance or resistance [20,21,22]. Gram-negative bacteria are intrinsically resistant to biocides due to their outer membrane, efflux pumps, and biofilm formation. Additionally, they can also acquire resistance genes via horizontal gene transfer. QAC resistance genes can be encoded on the chromosome (*emrE, mdfA, sugE, ydgE, ydgF*) or MGEs (*oqxA, oqxB, qacEΔ1, qacE, qacF/H/I, qacG, sugE*) such as plasmids, integrons, transposons, and integrative conjugative elements [21,23,24,25]. Proteins involved in QAC resistance belong to the small multidrug resistance (SMR) efflux family, with the exception of MdfA, a member of the major facilitator superfamily (MFS), and OqxAB from the resistance-nodulation-division (RND) family. Moreover, genes for these efflux pumps can be encoded on the same MGEs as antibiotic resistance genes, resulting in co-resistance or cross-resistance due to the same resistance mechanism [15,22,23,26]. The aim of our study was to determine the prevalence of QAC resistance genes and three classes of integrons in the collection of ESBL-EC isolated from the lower respiratory tract (LRT) samples, molecularly characterized for ST131 sequence type group and β-lactamase resistance genes.

## 2. Materials and Methods

### 2.1. Bacterial Isolates

*E. coli* isolates were obtained from LRT samples (sputa, tracheal aspirates, and bronchoalveolar lavages) between 2002 and 2022. All isolates were isolated and identified at the Institute of Microbiology and Immunology, Faculty of Medicine, University of Ljubljana (IMI), by using matrix-assisted laser desorption/ionization time-of-flight mass-spectrometry (MALDI TOF MS) (MBT COMPASS 4.1, Microflex, Bruker Daltonics, Bremen, Germany).

Furthermore, isolates were routinely tested for the phenotypic resistance to antimicrobial agents by disk diffusion assay. Results were interpreted according to Clinical and Laboratory Standards Institute (CLSI) [27] guidelines through 31 March 2014, and European Committee on Antimicrobial Susceptibility Testing (EUCAST) [28] guidelines since 1 April 2014. Extended-spectrum β-lactamase production was tested according to CLSI and EUCAST [29] recommendations in the aforementioned time frame. A total of 577 consecutive, unduplicated *E. coli* that were phenotypically and genotypically positive for ESBL and assigned to the ST131 group were molecularly analyzed.

### 2.2. Bacterial DNA Extraction and PCR Screening of QAC Resistance Genes

The boiling technique was used for bacterial DNA extraction [30]. Briefly, bacteria were harvested from 1.5 mL cultures by centrifugation and then resuspended in a total volume of 200 μL distilled sterile water and heated at 100 °C for 10 min. After a 10 min centrifugation, the supernatant-containing bacterial DNA was collected and used for all PCR reactions. All PCR amplifications were performed in a total volume of 25 μL containing 2 μL of the bacterial lysate, 12.5 μL of PCR Master mix (Thermo Fisher Scientific, Waltham, MA, USA), and each of the primers at a final concentration of 10 μM.

All 577 isolates were tested for the presence of QAC resistance genes and three classes of integrons using the specific primers and cycling conditions described in Table 1 [21,31,32,33,34].

### 2.3. Statistical Analysis

The Pearson Chi-square test was used to compare differences for categorical data by using IBM SPSS Statistics (version 25, IBM Analytics, Armonk, NY, USA). All tests were two-sided, and *p*-values < 0.05 were considered statistically significant. Spearman’s rho correlation was used to estimate the strength of the association between QAC resistance genes, integrons, sequence type groups, and β-lactamase genes. The correlation strength was categorized as very weak (0.00–0.19), weak (0.20–0.39), moderate (0.40–0.59), strong (0.60–0.79), and very strong (0.80–1.0).

## 3. Results

### 3.1. Prevalence of QAC Resistance Genes, Integrons, and ST131 Group in ESBL-EC Isolated from LRT Samples between 2002 and 2022

Among genes usually encoded on MGEs, we detected *qacEΔ1*, *sugE* (p), and *qacF/H/I* in 316/577 (54.6%), 5/577 (0.9%), and 2/577 (0.3%) isolates, respectively (Table 2 and Table S1 in Supplementary Materials for detailed information). Chromosome-encoded genes *mdfA*, *ydgE*, and *ydgF* were detected in all isolates; *sugE* (c) in 99% of isolates; and *emrE* in 76.9% of isolates. Genes *oqxA, oqxB, qacE,* and *qacG* were not detected. The comparison of QAC resistance genes between ST131 (n = 388; 67.2%) and non-ST131 (n = 189; 32.8%) groups revealed that *qacEΔ1* and *emrE* were statistically significant associated with clonal group ST131, while plasmid-encoded *sugE* and *qacF/H/I* were detected only in the non-ST131 group. In addition, 36.3% (210/577) of isolates were positive for class 1 integrons. The carriage of the *int1* was significantly correlated with clonal group ST131 assignment.

Of 445 *bla*_CTX-M-1_ positive isolates, one was positive for *qacF/H/I*, three for *sugE* (p), and 229 for *qacEΔ1,* while 212 isolates were negative for all MGE-encoded QAC resistance genes tested. Both of the two *bla*_CTX-M-2_ positive isolates carried *qacEΔ1*. Of 97 *bla*_CTX-M-9_ positive isolates, 65 were positive for *qacEΔ1*, and one isolate had a combination of *qacEΔ1* and *qacF/H/I*, while 31 isolates carried none of the MGE-encoded QAC resistance genes.

**Figure 1:**
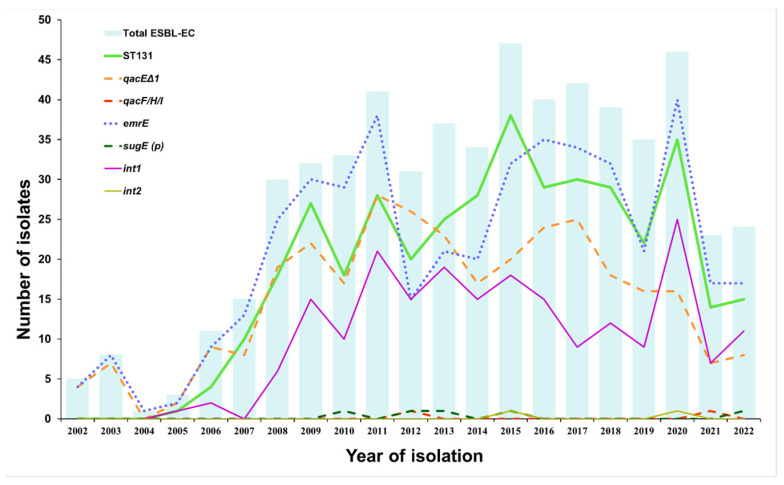
Distribution of QAC resistance genes, class 1 and 2 integrons, and ST131 clonal group among 577 ESBL-EC isolates over a 21-year period. Dashed lines represent the prevalence of MGE-encoded genes and dotted lines represent the prevalence of chromosome-encoded genes. QAC genes and integrons detected in all (*mdfA*, *sugE* (c), *ydgE*, *ydgF*) or none (*oqxA, oqxB, qacE, qacG, int3*) of the isolates are not shown. Data for Figure 1 are also presented in Table S2 in Supplementary Materials.

The distribution of isolates from LRT samples over a 21-year period revealed a lower number of ESBL-EC isolates after 2020, including isolates from the ST131 group (Figure 1 and Table S2). Accordingly, we also found a lower prevalence of *qacEΔ1* and *emrE* in 2021 and 2022.

### 3.2. Correlation between QAC Resistance Genes, Integrons, Sequence Type Group ST131, and β-Lactamase Genes Detected in ESBL-EC Isolates from LRT

**Figure 2:**
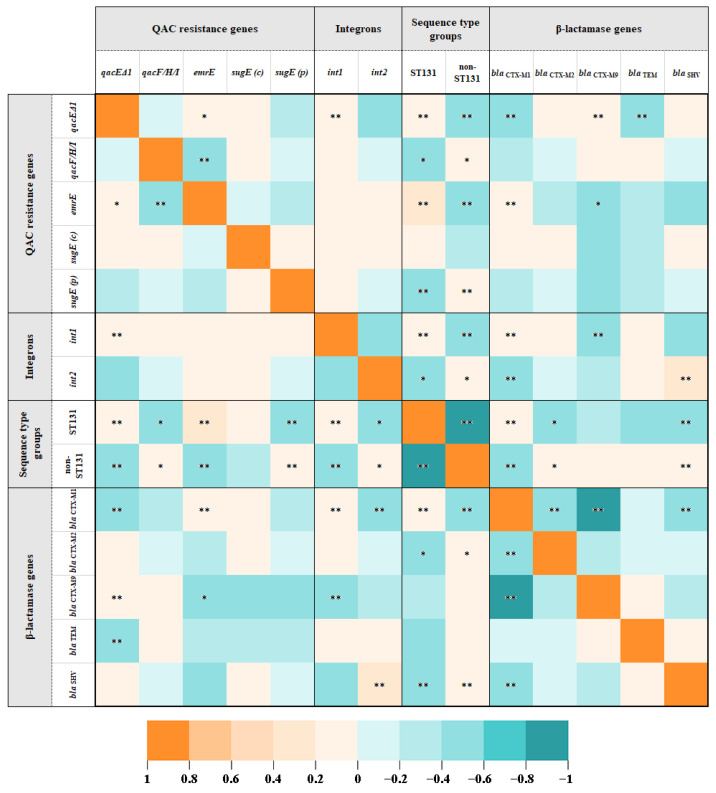
Heatmap showing color-coded correlation strength between QAC resistance genes, integrons, sequence type group ST131, and β-lactamase resistance genes detected in ESBL-EC isolates from LRT samples. The color values of the pairwise Spearman correlation coefficients (rho) in each cell are proportional to the strength of the correlations and range from orange (positive correlations) to turquoise (negative correlations). * represents statistically significant correlations at the 0.05 level and ** at the 0.01 level. QAC genes and integrons detected in all (*mdfA*, *ydgE*, *ydgF*) or none (*oqxA, oqxB, qacE, qacG, int3*) of the isolates are not shown.

The correlation matrix (Figure 2 and Table S3 in Supplementary Materials for detailed information) showed a statistically significant negative correlation between chromosome-encoded *emrE* and MGE-encoded *qacF/H/I* and a statistically significant positive correlation between *emrE* and MGE-encoded *qacEΔ1*. We also found a significant positive correlation between *qacEΔ1* and class 1 integrons (*p* < 0.001). Positive correlations were found between *int1* and ST131 and between *int2* and non-ST131. *emrE* and *qacEΔ1* were also weakly associated with the clonal group ST131, while *qacF/H/I* and *sugE* (p) were weakly associated with the non-ST131 group.

Analysis of QAC and β-lactamase resistance genes showed a positive correlation of *qacEΔ1* with *bla*_CTX-M-9_ and *emrE* with *bla*_CTX-M-1_. In addition, positive correlations were detected between *int1* and *bla*_CTX-M-1_ and between *int2* and *bla*_SHV_.

## 4. Discussion

Our study provides important insights into the QAC resistance profile of ESBL-producing *E. coli* isolated from LRT samples and its correlation with ST131, integrons, and β-lactamase resistance determinants.

Multidrug resistance, particularly to β-lactam and fluoroquinolone antimicrobials, is one of the most worrisome global health problems. Because of the different mechanisms of resistance, effective treatment options for bacterial infections are very limited. Murray and colleagues (2022) estimated that more than 1.5 million deaths in 2019 were associated with hospital- or community-acquired lower respiratory tract infections caused by antimicrobial-resistant bacteria [35]. Biocidal products, including antiseptics and disinfectants, are used to control and prevent the spread of pathogens and have been increasingly used since the SARS-CoV-2 outbreak. They are also widely used in hospitals to disinfect surfaces and instruments to prevent nosocomial infections. One of the most commonly used biocidal compounds are QACs. Several studies have reported efflux pumps as the main mechanism of resistance to biocidal agents. Additionally, they can also actively export other substances, such as antimicrobials and environmentally toxic compounds (e.g., heavy metals) [15,17,23,26,36].

In this study, we demonstrated that a variety of QAC resistance genes, especially chromosome-encoded, were present in ESBL-EC from LRT samples (Table 2). The most frequently detected QAC resistance gene profiles were *qacEΔ1*–*emrE*–*mdfA*–*sugE* (c)–*ydgE*–*ydgF* in 251 (43.5%) isolates, *emrE*–*mdfA*–*sugE* (c)–*ydgE*–*ydgF* in 187 (32.4%) isolates, *mdfA*–*sugE* (c)–*ydgE*–*ydgF* in 71 (12.3%) isolates, and *qacEΔ1*–*mdfA*–*sugE* (c)–*ydgE*–*ydgF* in 62 (10.7%) isolates. Similar QAC resistance profiles were detected in *E. coli* isolates from retail meat, with the most common genotypes being *emrE*–*mdfA*– *sugE* (c)–*ydgE*–*ydgF* (23–62%) and *mdfA*–*sugE* (c)–*ydgE*–*ydgF*(5–96%) [21,37,38].

In our study, the prevalence of chromosome-encoded genes *mdfA, sugE, ydgE,* and *ydgF* was nearly 100%, with the exception of *emrE*, which was detected in 76.9% of all isolates. The efflux pumps EmrE, SugE (c), YdgE, YdgF, and MdfA can export a variety of compounds, including QACs, to confer resistance [39]. However, unlike other genes, *ydgE* and *ydgF* must be co-expressed to confer resistance [21]. Accordingly, both genes were detected in all isolates (100%) in our study. In addition, we confirmed a statistically significant higher prevalence of *emrE* in isolates from the ST131 group than in the non-ST131 group (*p* < 0.001). Comparable results for the presence of chromosome-encoded genes have been obtained in other studies, while the prevalence of MGE-encoded genes varies between studies [21,25,40]. In a German study of 93 *E. coli* isolated from broiler farms, *sugE* (c), *ydgE*, *ydgF*, and *mdfA* were detected in all isolates tested, and *emrE* in 85% of isolates, while *qacEΔ1* and *sugE* (p) were detected in only nine and seven isolates, respectively [25].

*qacEΔ1*, the most prevalent QAC resistance gene in gram-negative bacteria, is a deletion mutation of *qacE*. Both genes confer resistance to QACs as well as to biguanide compounds and diamidines [41]. In this study, we showed that the frequency of QAC genes on MGEs was low, with the exception of *qacEΔ1* (54.6%). Moreover, the presence of *qacF/H/I* was confirmed in only two isolates, and *sugE* (p) in five isolates, while *oqxA*, *oqxB*, *qacE* and *qacG* were not detected. According to Zou et al. (2014), the most prevalent QAC resistance gene on MGEs was *qacEΔ1* (22.3%), followed by *sugE* (p) (6.8%) [21], while Zhang et al. (2016) detected *qacEΔ1* in 19.6% of isolates, with *qacF* (18%) being the second most prevalent gene [37]. A study by Sahin et al. (2022) on ESBL-EC isolates from chicken meat samples revealed a similar proportion of chromosome-encoded genes as in our study, but they found a higher prevalence of *qacF/H/I* (21.7%) and *sugE* (p) (6.7%), and a lower prevalence of *qacEΔ1* (20.0%) [40].

The majority of biocide-resistance studies are related to the food-processing environment, and only a few have been performed on clinical isolates of *E. coli*. A study of clinical isolates from hospitals in Iran revealed a similar proportion of *qacEΔ1* as our study (60.8% vs. 54.6%) but also detected *qacE* in 4.9% and a combination of both in 9.8% of 102 isolates [42]. In another study of clinical isolates, *qacEΔ1* was detected in all isolates tested (n = 150), and *qacE*, *qacF*, *qacG* in none [43]. In addition, differences were observed between ST131 (n = 388) and non-ST131 sequence type groups (n = 189). While we detected a statistically significant higher prevalence of *qacEΔ1*, *emrE,* and *int1* in the ST131 group, the *qacF/H/I*, *sugE* (p) and *int2* genes were statistically associated with the non-ST131 group.

Since *qacEΔ1* is often located on integrons, we screened all 577 isolates for the presence of the *int* gene specific for class 1, 2, and 3 integrons. Class 1 integrons can be localized on plasmids or transposons and are most commonly associated with antibiotic-resistant clinical isolates from the Enterobacteriaceae family, even ESBL-producing *E. coli*. Therefore, integron transfer may be critical for the spread of resistance genes through horizontal gene transfer [44,45,46]. Accordingly, the results of our study show a positive correlation between *qacEΔ1*, class 1 integrons, and *bla*_CTX-M-9_, confirming observations from previous studies, in which class 1 integrons and *qacEΔ1* were correlated with ESBL-EC isolates [44,45,46]. Surprisingly, our results also revealed that the prevalence of *qacEΔ1* was relatively low despite the enormous selection pressure due to the overuse of biocidal products in the SARS-CoV-2 pandemic.

Deus et al. (2017) located *qacE∆1*, *qacF*, *qacH,* and *sugE* (p) on large plasmids > 20 kb in ESBL-EC collected from humans and healthy broiler chickens, which can also carry *bla*_CTX-M_ [47] and can be transferred to other strains by conjugation [37,48]. The QAC tolerance determinants *qacE∆1* and *sugE* (p) were also found in close proximity to the antibiotic resistance genes *sul1* (sulfonamide resistance determinant) and *bla*_CMY-2_ [25]. Not only *qacE∆1* and *sul1*, but also *qacF* can be located in class 1 integrons, which can lead to the selection of strains with biocide- and antibiotic-resistant determinants [37].

This study demonstrates the widespread distribution of QAC resistance genes among ESBL-producing *E. coli* isolated from LRT samples and highlights the importance of appropriate use of biocidal products, especially in hospitals and food processing, to limit or prevent the spread of disinfectant and antibiotic resistance genes.

## 5. Conclusions

Biocides are used to prevent the spread of pathogens, not only in hospitals but also in food processing and domestic settings. However, their excessive and inappropriate use can lead to the selection of bacteria that are also cross-resistant to antimicrobials. Our study provides evidence for the presence of QAC resistance genes and integrons in clinical isolates of ESBL-producing *E. coli*, highlighting the potential transmission of antimicrobial resistance determinants via horizontal gene transfer. Furthermore, strains carrying both ESBL and QAC resistance genes have an advantage under the selection pressure in the patient receiving antimicrobials and also on medical instruments and/or surfaces in the clinical environment, allowing strains to persist and circulate in healthcare settings.

## Figures and Tables

**Table 1:** Primers and conditions for PCR amplification of QAC resistance genes.

Target	Primer (Sequence 5′→3′)	Amplification Conditions	PCR Product Size (bp)	Reference
QAC resistance genes				
*qacEΔ1*	qacEΔ1 F	95 °C—5 min	175	[21]
	(AATCCATCCCTGTCGGTGTT)	94 °C—30 s		
		56 °C—30 s 30×		
	qacEΔ1 R	72 °C—30 s		
	(CGCAGCGACTTCCACGATGGGGAT)	72 °C—7 min		
*qacE*	qacE F	95 °C—5 min	258	
	(AAGTAATCGCAACATCCG)	94 °C—30 s		
		50 °C—30 s 30×		
	qacE R	72 °C—30 s		
	(CTACTACACCACTAACTATGAG)	72 °C—7 min		
*qacF/H/I*	qacF/H/I F	95 °C—5 min	229	
	(GTCGTCGCAACTTCCGCACTG)	94 °C—30 s		
		60 °C—30 s 30×		
	qacF/H/I R	72 °C—30 s		
	(TGCCAACGAACGCCCACA)	72 °C—7 min		
*qacG*	qacG F ZOU	95 °C—5 min	122	
	(TCGCCTACGCAGTTTGGT)	94 °C—30 s		
		56 °C—30 s 30×		
	qacG R	72 °C—30 s		
	(AACGCCGCTGATAATGAA)	72 °C—7 min		
*emrE*	emrE F	95 °C—5 min	195	
	(TATTTATCTTGGTGGTGCAATAC)	94 °C—30 s		
		55 °C—30 s 30×		
	emrE R	72 °C—30 s		
	(ACAATACCGACTCCTGACCAG)	72 °C—7 min		
*mdfA*	mdfA F	95 °C—5 min	284	
	(GCATTGATTGGGTTCCTAC)	94 °C—30 s		
		55 °C—30 s 30×		
	mdfA R	72 °C—30 s		
	(CGCGGTGATCTTGATACA)	72 °C—7 min		
*sugE* (c)	sugE(c) F	95 °C—5 min	226	
	(CTGCTGGAAGTGGTATGGG)	94 °C—30 s		
		56 °C—30 s 30×		
	sugE(c) R	72 °C—30 s		
	(GCATCGGGTTAGCGGACT)	72 °C—7 min		
*sugE* (p)	sugE(p) F	95 °C—5 min	190	
	(GTCTTACGCCAAGCATTATCACTA)	94 °C—30 s		
		57 °C—30 s 30×		
	sugE(p) R	72 °C—30 s		
	(CAAGGCTCAGCAAACGTGC)	72 °C—7 min		
*ydgE*	ydgE F	95 °C—5 min	149	
	(GGCAATCGTGCTGGAAAT)	94 °C—30 s		
		55 °C—30 s 30×		
	ydgE R	72 °C—30 s		
	(CGACAGACAAGTCGATCCCT)	72 °C—7 min		
*ydgF*	ydgF F	95 °C—5 min	330	
	(TAGGTCTGGCTATTGCTACGG)	94 °C—30 s		
		55 °C—30 s 30×		
	ydgF R	72 °C—30 s		
	(GGTTCACCTCCAGTTCAGGT)	72 °C—7 min		
*oqxA*	oqxA F		671	[31]
	(GATCAGTCAGTGGGATAGTTT)	95 °C—5 min		
	oqxA r	95 °C—30 s		
	(TACTCGGCGTTAACTGATTA)	55 °C—30 s 30×		
*oqxB*	oqxBx F	72 °C—1.5 min	544	[32]
	(CCACCCTTAACTGATCCCTAA)	72 °C—10 min		
	oqxBx r			
	(CGCCAGCTCATCCTTCAC)			
Integrons				
*int1*	IntI1-F	95 °C—5 min	483	[33]
	(GGTCAAGGATCTGGATTTCG)	94 °C—30 s		
		62 °C—30 s 30×		
	IntI1-R	72 °C—1 min		
	(ACATGCGTGTAAATCATCGTC)	72 °C—7 min		
*int2*	IntI2-F	95 °C—5 min	789	
	(CACGGATATGCGACAAAAAGGT)	94 °C—30 s		
		62 °C—30 s 30×		
	IntI2-R	72 °C—1 min		
	(GTAGCAAACGAGTGACGAAATG)	72 °C—7 min		
*int3*	IntI3-F	95 °C—5 min	600	[34]
	(AGTGGGTGGCGAATGAGTG)	94 °C—30 s		
		60 °C—30 s 30×		
	IntI3-R	72 °C—1 min		
	(TGTTCTTGTATCGGCAGGTG)	72 °C—7 min		

**Table 2:** Prevalence of QAC resistance genes and class 1, 2, and 3 integrons in relation to ST131 affiliation among 577 ESBL-EC isolates from LRT samples with corresponding Pearson Chi-square and *p*-values.

QAC Resistance Genes	Total ESBL	ST131	Non-ST131	Pearson Chi-Square Value	*p*-Value ^1^
	N = 577 (100%)	N = 388 (100%)	N = 189 (100%)	(df 1)	
	n (%)	n (%)	n (%)		
MGE-encoded genes					
*oqxA*	0 (0%)	0 (0%)	0 (0%)		
*oqxB*	0 (0%)	0 (0%)	0 (0%)		
*qacEΔ1*	316 (54.6%)	230 (59.1%)	86 (45.3%)	9.8	0.002
*qacE*	0 (0%)	0 (0%)	0 (0%)		
*qacF/H/I*	2 (0.3%)	0 (0%)	2 (1.1%)	4.1	0.042
*qacG*	0 (0%)	0 (0%)	0 (0%)		
*sugE* (p)	5 (0.9%)	0 (0%)	5 (2.6%)	10.4	0.001
Chromosome-encoded genes					
*emrE*	443 (76.9%)	340 (87.7%)	103 (54.7%)	78.2	<0.001
*mdfA*	577 (100%)	388 (100%)	189 (100%)		
*sugE* (c)	571 (99%)	385 (99.2%)	186 (98.4%)	0.8	0.366
*ydgE*	577 (100%)	388 (100%)	189 (100%)		
*ydgF*	577 (100%)	388 (100%)	189 (100%)		
Integrons					
*int1*	210 (36.3%)	157 (40.4%)	53 (27.9%)	8.7	0.004
*int2*	2 (0.3%)	0 (0%)	2 (1.1%)	4.1	0.042
*int3*	0 (0%)	0 (0%)	0 (0%)		

^1^ *p*-values (ST131 vs. non-ST131) calculated by Chi-square test are shown. *p*-values < 0.05 were considered statistically significant.

## Data Availability

The data supporting the results of this study are available in the Supplementary Materials or upon reasonable request from the corresponding author (J.A.A.).

## Supplementary Materials

The following supporting information can be downloaded at: https://www.mdpi.com/article/10.3390/tropicalmed8050273/s1, Table S1. Research raw data; Table S2. QAC resistance genes distribution among ESBL-producing *E. coli* between 2002 and 2022; Table S3. Spearman correlation coefficients (rho) between QAC resistance genes, sequence type groups, phylogenetic groups, β-lactamase, and PMQR genes detected in ESBL-EC isolates from LRT.

## Abbreviations

CLSI: Clinical and Laboratory Standards Institute; ESBL: Extended-spectrum β-lactamase; ESBL-EC: Extended-spectrum β-lactamase-producing *E. coli*; EUCAST: European Committee on Antimicrobial Susceptibility Testing; IMI: Institute of Microbiology and Immunology, Faculty of Medicine, University of Ljubljana; LRT: Lower respiratory tract; MALDI TOF MS: Matrix-assisted laser desorption/ionization time-of-flight mass-spectrometry; MGE: Mobile genetic element; PMQR: Plasmid-mediated quinolone resistance; QACs: Quaternary ammonium compounds.

## References

[1] Poolman J.T., Wacker M. (2016). Extraintestinal Pathogenic *Escherichia coli*, a Common Human Pathogen: Challenges for Vaccine Development and Progress in the Field. J. Infect. Dis..

[2] Rogers B.A., Sidjabat H.E., Paterson D.L. (2011). *Escherichia coli* O25b-ST131: A pandemic, multiresistant, community-associated strain. J. Antimicrob. Chemother..

[3] Sarowska J., Futoma-Koloch B., Jama-Kmiecik A., Frej-Madrzak M., Ksiazczyk M., Bugla-Ploskonska G., Choroszy-Krol I. (2019). Virulence factors, prevalence and potential transmission of extraintestinal pathogenic *Escherichia coli* isolated from different sources: Recent reports. Gut Pathog..

[4] Massella E., Giacometti F., Bonilauri P., Reid C.J., Djordjevic S.P., Merialdi G., Bacci C., Fiorentini L., Massi P., Bardasi L. (2021). Antimicrobial Resistance Profile and ExPEC Virulence Potential in Commensal *Escherichia coli* of Multiple Sources. Antibiotics.

[5] Biran D., Ron E.Z. (2018). Extraintestinal Pathogenic *Escherichia coli*. Curr. Top. Microbiol. Immunol..

[6] Smith J.L., Fratamico P.M., Gunther N.W. (2007). Extraintestinal pathogenic *Escherichia coli*. Foodborne Pathog. Dis..

[7] Pitout J.D. (2012). Extraintestinal Pathogenic *Escherichia coli*: A Combination of Virulence with Antibiotic Resistance. Front. Microbiol..

[8] Fagerström A., Mölling P., Khan F.A., Sundqvist M., Jass J., Söderquist B. (2019). Comparative distribution of extended-spectrum beta-lactamase-producing *Escherichia coli* from urine infections and environmental waters. PLoS ONE.

[9] Nicolas-Chanoine M.H., Bertrand X., Madec J.Y. (2014). *Escherichia coli* ST131, an intriguing clonal group. Clin. Microbiol. Rev..

[10] Azargun R., Sadeghi M.R., Soroush Barhaghi M.H., Samadi Kafil H., Yeganeh F., Ahangar Oskouee M., Ghotaslou R. (2018). The prevalence of plasmid-mediated quinolone resistance and ESBL-production in Enterobacteriaceae isolated from urinary tract infections. Infect. Drug Resist..

[11] García-Fulgueiras V., Bado I., Mota M.I., Robino L., Cordeiro N.F., Varela A., Algorta G., Gutkind G., Ayala J.A., Vignoli R. (2011). Extended-spectrum β-lactamases and plasmid-mediated quinolone resistance in enterobacterial clinical isolates in the paediatric hospital of Uruguay. J. Antimicrob. Chemother..

[12] Bado I., Gutiérrez C., García-Fulgueiras V., Cordeiro N.F., Araújo Pirez L., Seija V., Bazet C., Rieppi G., Vignoli R. (2016). CTX-M-15 in combination with *aac(6′)-Ib-cr* is the most prevalent mechanism of resistance both in *Escherichia coli* and *Klebsiella pneumoniae*, including *K. pneumoniae* ST258, in an ICU in Uruguay. J. Glob. Antimicrob. Resist..

[13] Machuca J., Ortiz M., Recacha E., Diaz-De-Alba P., Docobo-Perez F., Rodriguez-Martinez J.M., Pascual A. (2016). Impact of *AAC(6′)-Ib-cr* in combination with chromosomal-mediated mechanisms on clinical quinolone resistance in *Escherichia coli*. J. Antimicrob. Chemother..

[14] WHO WHO Publishes List of Bacteria for Which New Antibiotics Are Urgently Needed. https://www.who.int/news/item/27-02-2017-who-publishes-list-of-bacteria-for-which-new-antibiotics-are-urgently-needed.

[15] Chen B., Han J., Dai H., Jia P. (2021). Biocide-tolerance and antibiotic-resistance in community environments and risk of direct transfers to humans: Unintended consequences of community-wide surface disinfecting during COVID-19?. Environ. Pollut..

[16] Jones I.A., Joshi L.T. (2021). Biocide Use in the Antimicrobial Era: A Review. Molecules.

[17] Maillard J.-Y. (2005). Antimicrobial biocides in the healthcare environment: Efficacy, usage, policies, and perceived problems. Ther. Clin. Risk Manag..

[18] Vincent J.L. (2003). Nosocomial infections in adult intensive-care units. Lancet.

[19] Gerba C.P. (2015). Quaternary ammonium biocides: Efficacy in application. Appl. Environ. Microbiol..

[20] Meade E., Slattery M.A., Garvey M. (2021). Biocidal Resistance in Clinically Relevant Microbial Species: A Major Public Health Risk. Pathogens.

[21] Zou L., Meng J., McDermott P.F., Wang F., Yang Q., Cao G., Hoffmann M., Zhao S. (2014). Presence of disinfectant resistance genes in *Escherichia coli* isolated from retail meats in the USA. J. Antimicrob. Chemother..

[22] Maertens H., Demeyere K., De Reu K., Dewulf J., Vanhauteghem D., Van Coillie E., Meyer E. (2020). Effect of subinhibitory exposure to quaternary ammonium compounds on the ciprofloxacin susceptibility of *Escherichia coli* strains in animal husbandry. BMC Microbiol..

[23] Wand M.E., Hurst C.J. (2017). Bacterial Resistance to Hospital Disinfection. Modeling the Transmission and Prevention of Infectious Disease.

[24] Mc Carlie S., Belter B., Van Der Walt B., Bragg R., Guillermo T.-I. (2022). Molecular Tools for the Study of Resistance to Disinfectants. The Global Antimicrobial Resistance Epidemic.

[25] Roedel A., Vincze S., Projahn M., Roesler U., Robé C., Hammerl J.A., Noll M., Al Dahouk S., Dieckmann R. (2021). Genetic but No Phenotypic Associations between Biocide Tolerance and Antibiotic Resistance in *Escherichia coli* from German Broiler Fattening Farms. Microorganisms.

[26] Buffet-Bataillon S., Tattevin P., Maillard J.Y., Bonnaure-Mallet M., Jolivet-Gougeon A. (2016). Efflux pump induction by quaternary ammonium compounds and fluoroquinolone resistance in bacteria. Future Microbiol..

[27] CLSI (2019). Performance Standards for Antimicrobial Susceptibility Testing.

[28] EUCAST Breakpoint Tables for Interpretation of MICs and Zone Diameters. Versions 4.0 to 9.0. https://www.eucast.org/clinical_breakpoints/.

[29] EUCAST Guidelines for Detection of Resistance Mechanisms and Specific Resistances of Clinical and/or Epidemiological Importance. Version 2.0. pp. 1–43. http://www.eucast.org/resistance_mechanisms/.

[30] Le Bouguenec C., Archambaud M., Labigne A. (1992). Rapid and specific detection of the *pap*, *afa*, and *sfa* adhesin-encoding operons in uropathogenic *Escherichia coli* strains by polymerase chain reaction. J. Clin. Microbiol..

[31] Hansen L.H., Sørensen S.J., Jørgensen H.S., Jensen L.B. (2005). The prevalence of the OqxAB multidrug efflux pump amongst olaquindox-resistant *Escherichia coli* in pigs. Microb. Drug Resist..

[32] Ni Q., Tian Y., Zhang L., Jiang C., Dong D., Li Z., Mao E., Peng Y. (2016). Prevalence and quinolone resistance of fecal carriage of extended-spectrum β-lactamase-producing *Escherichia coli* in 6 communities and 2 physical examination center populations in Shanghai, China. Diagn. Microbiol. Infect. Dis..

[33] Mazel D., Dychinco B., Webb V.A., Davies J. (2000). Antibiotic resistance in the ECOR collection: Integrons and identification of a novel aad gene. Antimicrob. Agents Chemother..

[34] Goldstein C., Lee M.D., Sanchez S., Hudson C., Phillips B., Register B., Grady M., Liebert C., Summers A.O., White D.G. (2001). Incidence of class 1 and 2 integrases in clinical and commensal bacteria from livestock, companion animals, and exotics. Antimicrob. Agents Chemother..

[35] Murray C.J.L., Ikuta K.S., Sharara F., Swetschinski L., Robles Aguilar G., Gray A., Han C., Bisignano C., Rao P., Wool E. (2022). Global burden of bacterial antimicrobial resistance in 2019: A systematic analysis. Lancet.

[36] Tong C., Hu H., Chen G., Li Z., Li A., Zhang J. (2021). Disinfectant resistance in bacteria: Mechanisms, spread, and resolution strategies. Environ. Res..

[37] Zhang A., He X., Meng Y., Guo L., Long M., Yu H., Li B., Fan L., Liu S., Wang H. (2016). Antibiotic and Disinfectant Resistance of *Escherichia coli* Isolated from Retail Meats in Sichuan, China. Microb. Drug Resist..

[38] Sun Y., Hu X., Guo D., Shi C., Zhang C., Peng X., Yang H., Xia X. (2019). Disinfectant Resistance Profiles and Biofilm Formation Capacity of *Escherichia coli* Isolated from Retail Chicken. Microb. Drug Resist..

[39] Pal C., Bengtsson-Palme J., Rensing C., Kristiansson E., Larsson D.G. (2014). BacMet: Antibacterial biocide and metal resistance genes database. Nucleic Acids Res..

[40] Sahin S., Mogulkoc M.N., Kürekci C. (2022). Disinfectant and heavy metal resistance profiles in extended spectrum β-lactamase (ESBL) producing *Escherichia coli* isolates from chicken meat samples. Int. J. Food Microbiol..

[41] Vijayakumar R., Sandle T. (2019). A review on biocide reduced susceptibility due to plasmid-borne antiseptic-resistant genes-special notes on pharmaceutical environmental isolates. J. Appl. Microbiol..

[42] Habibollah-Pourzereshki N., Peymani A., Keshavarz-Saleh F. (2020). The Emergence of Quaternary Ammonium Compounds Resistance in *Escherichia coli* Isolated from Hospitals of Qazvin, Iran. Infect. Disord. Drug Targets.

[43] Hadadi F., Ghaznavirad E., Almasi-Hashiani A., Abtahi H. (2019). Detection of *qacEΔ1*, *qacG*, *qacE*, *qacF* resistance genes in *Escherichia coli* producing broad-spectrum beta-lactamases to benzalkonium chloride. J. Babol Univ. Med. Sci..

[44] Leverstein-van Hall M.A., Blok H.E.M., Donders A.R.T., Paauw A., Fluit A.C., Verhoef J. (2003). Multidrug Resistance among Enterobacteriaceae Is Strongly Associated with the Presence of Integrons and Is Independent of Species or Isolate Origin. J. Infect. Dis..

[45] Martinez-Freijo P., Fluit A.C., Schmitz F.J., Grek V.S., Verhoef J., Jones M.E. (1998). Class I integrons in Gram-negative isolates from different European hospitals and association with decreased susceptibility to multiple antibiotic compounds. J. Antimicrob. Chemother..

[46] Sun J., Zheng F., Wang F., Wu K., Wang Q., Chen Q., Yu S., Rui Y. (2013). Class 1 integrons in urinary isolates of extended-spectrum β-lactamase-producing *Escherichia coli* and *Klebsiella pneumoniae* in Southern China during the past five years. Microb. Drug Resist..

[47] Deus D., Krischek C., Pfeifer Y., Sharifi A.R., Fiegen U., Reich F., Klein G., Kehrenberg C. (2017). Comparative analysis of the susceptibility to biocides and heavy metals of extended-spectrum β-lactamase-producing *Escherichia coli* isolates of human and avian origin, Germany. Diagn. Microbiol. Infect. Dis..

[48] Pal C., Bengtsson-Palme J., Kristiansson E., Larsson D.G.J. (2015). Co-occurrence of resistance genes to antibiotics, biocides and metals reveals novel insights into their co-selection potential. BMC Genom..

